# The role of transcriptional coactivator TAZ in gliomas

**DOI:** 10.18632/oncotarget.12625

**Published:** 2016-10-13

**Authors:** Weijie Li, Shicai Dong, Wei Wei, Guangxiu Wang, Anling Zhang, Peiyu Pu, Zhifan Jia

**Affiliations:** ^1^ Department of Neurosurgery, Tianjin Medical University General Hospital, Tianjin, P.R. China; ^2^ Department of Neurosurgery, Tianjin Medical University General Hospital, Tianjin, People's Republic of China; ^3^ Key Laboratory of Post-trauma Neuro-repair and Regeneration in Central Nervous System, Ministry of Education, Tianjin, P.R. China; ^4^ Tianjin Key Laboratory of Injuries, Variations and Regeneration of Nervous System, Tianjin, P.R. China

**Keywords:** glioma, Hippo pathway, TAZ, TEAD4, proliferation

## Abstract

The transcriptional coactivator with PDZ-binding motif (TAZ) is one of the important downstream effectors of Hippo pathway. In this study, the potential implication of TAZ in gliomagenesis was explored. TAZ expression was identified to be upregulated in glioma specimens and positively correlated with tumor grade. Meanwhile, its expression in nucleus was increased more significantly with the ascending order of tumor grade. Knocking down TAZ inhibited glioma cell proliferation, invasion and promoted apoptosis. Conversely, enforced upregulation of TAZ promoted proliferation, invasion of glioma cells, and suppressed apoptosis *in vitro*. When orthotopic glioblastoma mouse model implanted with TAZ knocked down cells, glioma growth was inhibited and survival period was prolonged. Expression of Ki67, MMP-9, Cyclin D1, Bcl-2 and C-myc was varied in accordance with the level of TAZ in glioma cell. The biomarkers of EMT (epithelial-mesenchymal transition), vimentin and N-cadherin, were downregulated when TAZ was suppressed. Using Co-immunoprecipitation TAZ was identified to bind to TEAD4. Therefore, our findings indicate that TAZ is overexpressed in glioma and translocated more into nucleus in high grade glioma. TAZ is involved in gliomagenesis by promoting glioma growth and may benefit to EMT progression. This result suggests that TAZ serves as a potential target for the treatment of glioma.

## INTRODUCTION

Glioma is the most common primary intracranial tumor, characterized by diffusely infiltrative growth and highly cellular heterogeneity associated with therapeutic resistance.

The Hippo pathway was firstly discovered in Drosophila, and plays a key role in the regulation of organ size by controlling cell proliferation and apoptosis [1–3]. The Hippo pathway is conserved from fly to mammal. TAZ (transcriptional co-activator with PDZ-binding motif) and its paralog, YAP (Yes-associated protein), are downstream effectors of Hippo pathway. The Hippo pathway inactivates TAZ by a series of phosphorylation events. Phosphorylated TAZ (p-TAZ) is maintained in the cytoplasm by interaction with 14-3-3 proteins or degraded via proteasome-targeted degradation. When Hippo pathway is inhibited, YAP and TAZ are translocated to the nucleus as transcriptional co-activators, TAZ binds with other transcription factors, such as TEADs, PPAR, RUNX2, etc and stimulates the transcription of its target genes to promote cell proliferation and EMT [4–6].

In recent years, aberrant expression of TAZ has been identified in a variety of cancers, such as breast cancer, liver cancer, colorectal cancer, lung cancer and hepatocellular carcinoma etc [7–10]. In non-small cell lung cancer (NSCLC) overall survival of patients with TAZ negative tumor was significantly prolonged compared to those with TAZ positive tumor [11]. Very few studies on TAZ alteration in gliomas have been reported. TAZ overexpression was detected in glioma samples only by immunohistochemistry [12]. Thus, we aim to study of the expression of TAZ in gliomas, and the effect of TAZ on growth of glioma cells *in vitro* and *in vivo*. We believe the result will be helpful for understanding the contribution of TAZ to tumorigenesis of glioma.

## RESULTS

### TAZ is overexpressed in glioma specimens and correlates with tumor grade

**Figure 1:**
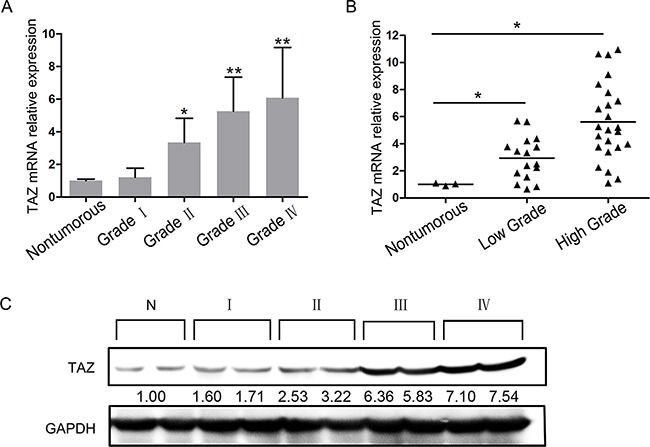
Expression of TAZ in glioma specimens Glioma specimens consisted of 3 cases of pilocytic astrocytoma (Grade I), 8 cases of astrocytoma (Grade II), 5 cases of oligodendroglioma (Grade II), 6 cases of anaplastic astrocytoma (Grade III), 3 cases of anaplastic oligodendroglioma (Grade III) and 16 cases of glioblastoma (GBM, Grade IV). **A.** Expression of TAZ in grade I~IV glioma specimens detected by RT-PCR. **B.** Expression of TAZ in low grade and high grade glioma specimens detected by RT-PCR. **C.** Expression of TAZ in grade I~IV glioma specimens detected by Western blotting.

We defined the TAZ expression status in 41 glioma specimens and 3 nontumorous brain tissues. Quantitative PCR indicated that TAZ expression in 38 cases of 41 glioma tissues (92.68%) was increased than that in nontumorous brain tissues, and positively correlated with tumor grade (P<0.05, Figure 1A, 1B). TAZ expression level was significantly higher in grade III/IV than that of grade I/II gliomas (Figure 1B). TAZ protein expression was detected in the same specimens by Western blotting, the result also showed that TAZ was expressed at higher levels in glioma specimens (WHO II to IV) compared with nontumorous brain tissues, and the expression level was also elevated with the ascending order of tumor grade (Figure 1C).

### TAZ expression and its subcellular localization in glioma specimens

**Figure 2:**
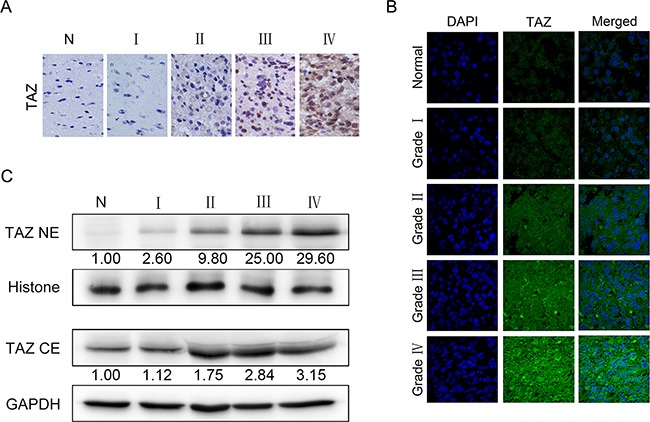
Localization and expression of TAZ in glioma specimens **A.** Localization and expression of TAZ in glioma specimens detected by immunohistochemical staining (×200). **B**. Localization and expression of TAZ in glioma specimens detected by immunofluorescence staining (×1000). **C.** Expression of TAZ in cytoplasm and nucleus of glioma specimens detected by Western blotting. TAZ expression level of nucleus in grade I, II, III, IV gliomas is 2.6, 9.8, 25, 29.6 fold higher than that in nontumorous brain, respectively, whereas TAZ expression in cytoplasm in grade I, II, III, IV gliomas is 1.12, 1.75, 2.84, 3.15 fold higher than that in nontumorous brain, respectively. The results demonstrate that the expression level of TAZ in cell nucleus increased more significantly with the ascending order of tumor grade.

Western blotting, Immunohistochem (IHC) and immunoflurosence (IF) staining were employed to further explore the subcellular localization of TAZ in glioma specimens. IHC showed that TAZ was similarly overexpressed in gliomas and positively correlated with tumor grade (Figure 2A). TAZ expression was restricted to cytoplasm in low grade tumors, whereas it could be detected in both nucleus and cytoplasm in high grade tumors. Meanwhile, TAZ staining intensity and the number of positive staining cells increased with the elevation of tumor grade (Figure 2A). IF results also indicated that TAZ expression localized in both nucleus and cytoplasm in high grade glioma specimens, the TAZ fluorescence intensity was enhanced along with the increase of tumor grade especially in the nucleus (Figure 2B). Additionally, Western blotting of cytoplasm and nucleus protein separately indicated that TAZ expression in nucleus was also positively correlated with tumor grade. Although TAZ expression in cytoplasm exhibited the same increasing tendency but not so significant as that in nucleus. The TAZ upregulation in high grade tumor compared to control is more obvious in nucleus than in cytoplasm. There was almost no detectable expression of TAZ in cell nucleus of nontumorous brain tissues, which further confirmed the IF results (Figure 2C).

### TAZ promotes glioma cell proliferation

**Figure 3:**
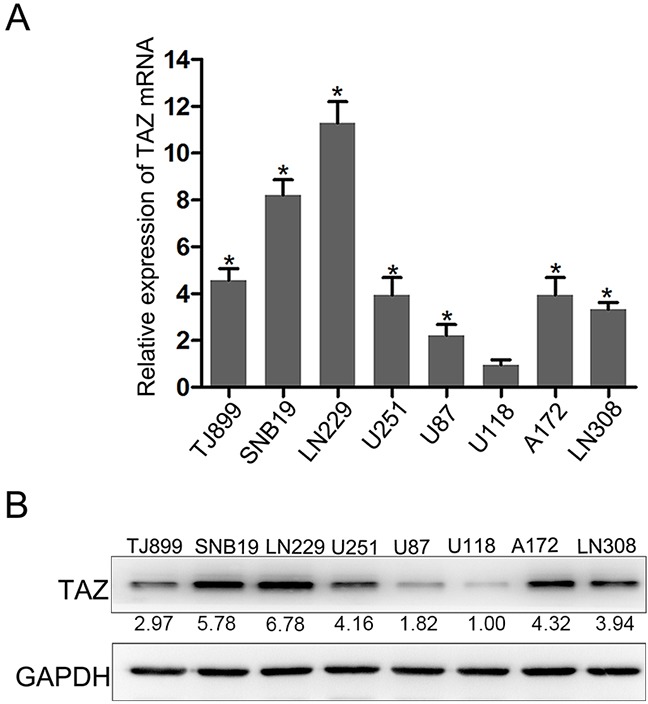
Expression of TAZ in glioma cell lines **A.** Expression of TAZ in glioma cell lines detected by RT-PCR. **B.** Expression of TAZ in glioma cell lines detected by Western blotting, whereas TAZ expression in LN229, SNB19, A172, U251, LN308, TJ899, U87 cell is 6.78, 5.78, 4.32, 4.16, 3.94, 2.97, 1.82 fold higher than that in U118 cell.

**Figure 4:**
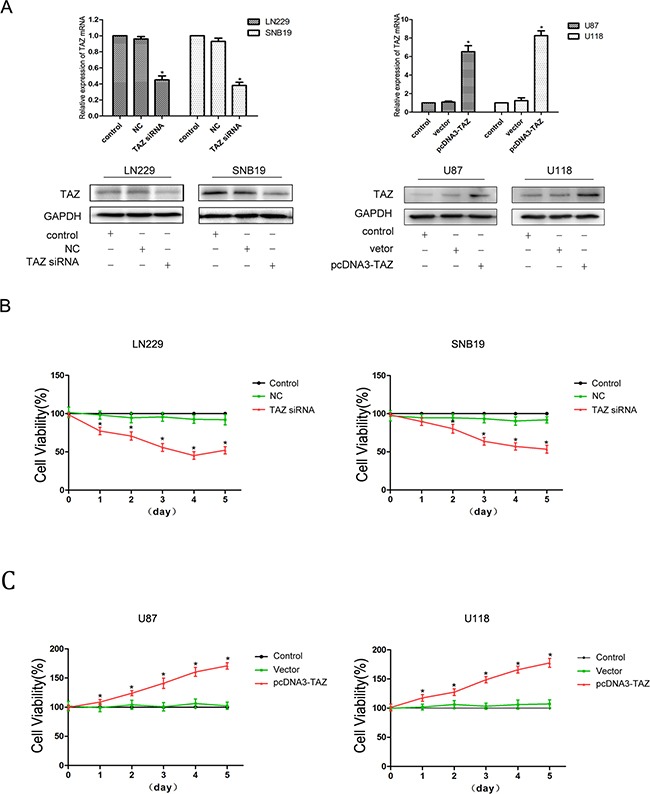
TAZ promotes proliferation of glioma cells **A.** TAZ expression was knocked down by transfection of TAZ siRNA, and TAZ expression was upregulated by transfection of pcDNA3-TAZ detected by RT-PCR and Western blotting in glioma cells **B.** Glioma cell proliferation was inhibited when TAZ was knocked down (*: compared with control group, P<0.05). **C.** Glioma cell viability was enhanced when TAZ was upregulated (*: compared with control group, P<0.05).

We have evaluated the expression of TAZ in 8 glioma cell lines (TJ899, SNB19, LN229, U251, U87, U118, A172, LN308) by Quantitative PCR (Figure 3). In LN229 and SNB19 cells, expression of TAZ was upregulated significantly, while in U118 and U87, TAZ level was lower than other glioma cell lines. So TAZ siRNA was transfected to LN229 and SNB19 cell lines, and pcDNA3-TAZ was transfected to U118 and U87 cells for further clarifying the effect of TAZ on cell behavior. As shown by Western blotting and RT-PCR (Figure 4A), the expression of TAZ was knocked down in LN229 and SNB19 cells and upregulated in U118 and U87 cells after transfection. The growth in these two group cells was examined consecutively up to 5 days after transfection. During the observation period, cell growth was decreased in TAZ knocked down cells, and increased in TAZ overexpressed cells (Figure 4B, 4C)

### TAZ speeds up cell cycle progression

**Figure 5:**
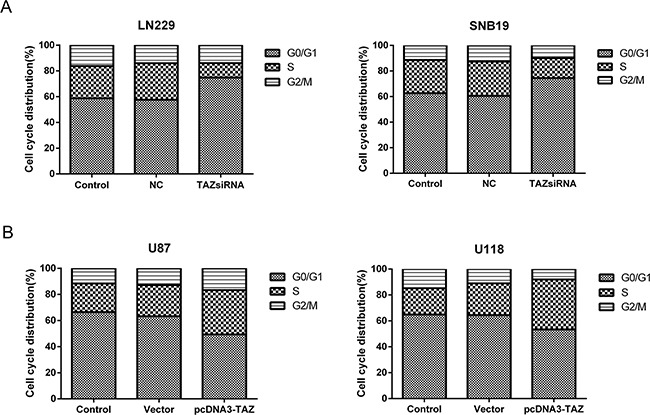
TAZ speeds up cell cycle progression **A.** Cell population in S phase was decreased and Cell cycle was arrested at G0/G1 phase in LN229 and SNB19 cells when TAZ was knocked down (P<0.05). **B.** Cell population in S phase was increased while G0/G1 phase reduced significantly in PCDNA3-TAZ transfected U87 and U118 Cells (P<0.05), suggesting that cell cycle progression was promoted.

The cell-cycle kinetics was analyzed using flow cytometry. Compared with control group, treatment with TAZ siRNA resulted in the cell population arrested in G0/G1 phase (p<0.05) (Figure 5A). Conversely, cell population was reduced in G0/G1 phase in TAZ transfected cells (P<0.05). This evidence suggested that cell cycle was promoted by TAZ (Figure 5B)

### TAZ enhances invasion and migration of glioma cells

**Figure 6:**
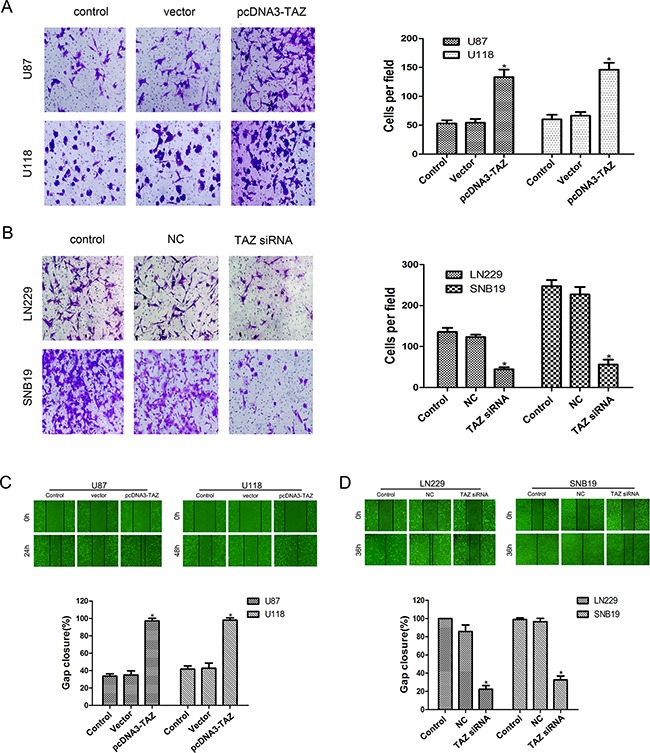
TAZ enhances invasion and migration of glioma cells **A.** Invasion of U118 and U87 cells transfected with pcDNA3-TAZ was enhanced as shown in Transwell assay. **B.** Invasion of LN229 and SNB19 cells transfected with TAZ siRNA was attenuated in Transwell assay (*Compared with control group, p<0.05). **C.** Migration of LN229 and SNB19 cells transfected with TAZ siRNA was inhibited as detected by Scratch assay. **D.** Enhanced migration of U118 and U87 cells transfected with pcDNA3-TAZ was detected by Scratch assay (*Compared with control group, p<0.05).

In transwell invasion assay, the cells that had invaded to the lower surface of the filter membrane were counted. As compared to the control counterpart, the number of invasive cells in pcDNA3-TAZ transfected U118 and U87 cells was increased (Figure 6A), whereas the number of invasive cells in the LN229 and SNB19 cells transfected with TAZ siRNA was decreased (Figure 6B)

Using scratch assay to examine cell migration *in vitro*, we found that after 24 hr of incubation, decreased mobility was observed for TAZ siRNA treated cells (Figure 6C), while cell migration was increased in pcDNA3-TAZ transfected cells (Figure 6D).

### TAZ suppresses apoptosis of glioma cells

**Figure 7:**
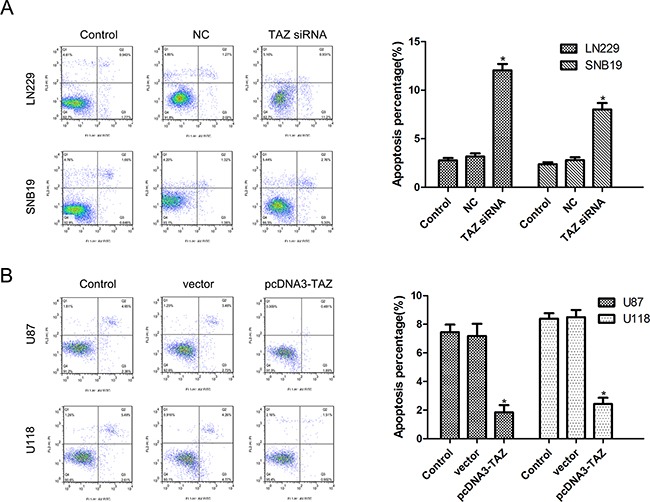
TAZ suppresses apoptosis of glioma cells detected by Annexin V staining **A.** Apoptosis was induced in TAZ siRNA cell group (*Compared with control cell group, p<0.05). **B.** Apoptosis was inhibited in pcDNA3-TAZ cell group (*Compared with control cell group, p<0.05).

The effect of TAZ on apoptosis was analyzed by flow cytometry with Annexin V and PI double staining. As shown in the histograms (Figure 7), downregulation of TAZ in SNB19 and LN229 cells resulted in an increase of apoptotic cells, while upregulation of TAZ in U118 and U87 cells led to a significant decrease of apoptotic cells, indicating that TAZ suppressed apoptosis in glioma cells.

### Knocking down TAZ inhibits glioma growth *in vivo*

**Figure 8:**
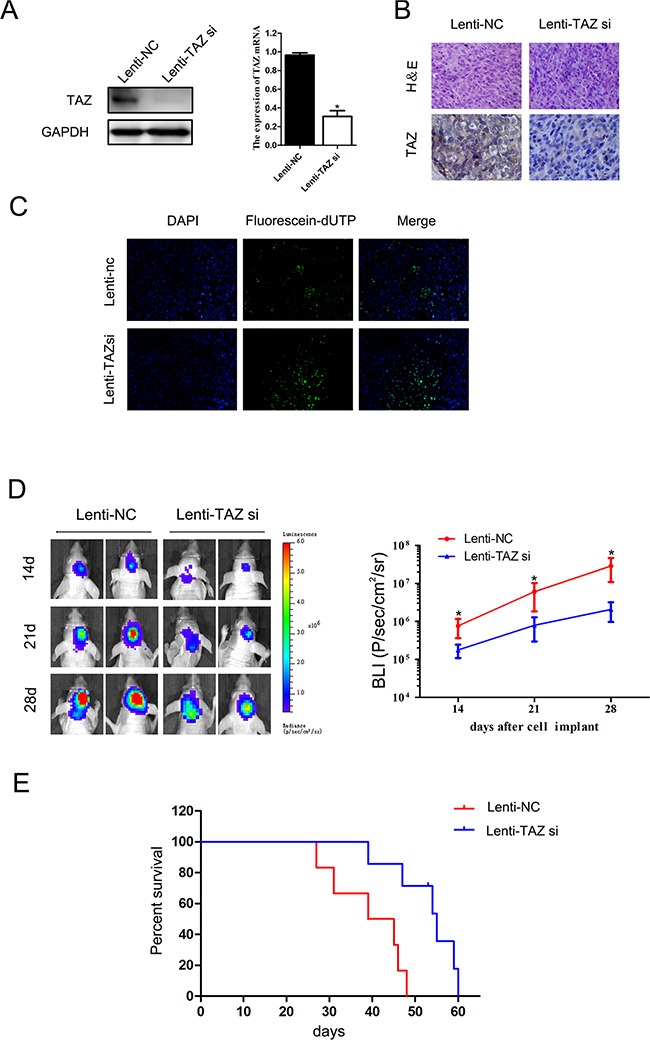
The effect of TAZ on glioma growth *in vivo* **A.** Expression of TAZ in LN229 cell was knocked down after Luc-TAZ si-Lentivirus transfection detected by Western blotting and RT-PCR. **B.** TAZ expression was suppressed in tumor samples of Lenti-TAZ si group detected by IHC. **C.** TAZ si induced apoptosis in xenograft specimen detected by TUNEL method. **D.** BLI signals in TAZ si xenografts were weaker than those in Lenti-NC xenografts. **E.** The survival of six mice in TAZ si group (39, 47, 54, 55, 59, 60 days, respectively) was longer than that in Lenti-NC group (27, 31, 39, 45, 46, 48 days, respectively), the survival of mice in lenti-TAZ si group was significantly prolonged (*p*<0.05).

After transfected with Luc-TAZ si-Lentivirus, expression of TAZ in LN229 cell was knocked down (Figure 8A). In xenograft tumor samples of Lenti-TAZ si group, expression of TAZ was downregulated detected by IHC (Figure 8B).

Our *in vivo* study found that bioluminescence imaging (BLI) signals were weaker in Lenti-TAZ si group than those in Lenti-NC group (Figure 8D) during the regular observation period. Meanwhile, compared the survival of nude mice in Lenti-NC group (mean survival: 39.2±8.5 days) with that in TAZ si group (mean survival: 52.3±8.0 days), the survival of mice in lenti-TAZ si group was significantly prolonged (p<0.05) (Figure 8C).

The number of apoptotic cells in orthotopic xenografts implanting with LN229 cells transfected with lenti-TAZ si was also markedly increased as detected by the TUNEL staining (Figure 8E). This result of *in vivo* study was coincident with that of *in vitro*.

### Regulation of TAZ on the biomarkers relevant to tumor cell biological behavior

**Figure 9:**
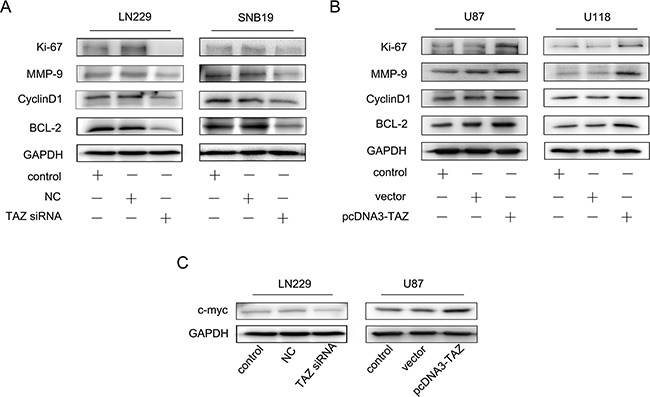
Effect of TAZ on expression of Ki67, MMP-9, Cyclin D1, Bcl-2 *in vitro* **A.** Expression of Ki67, MMP-9, Cyclin D1, Bcl-2 was suppressed when TAZ was knocked down. **B.** Expression of Ki67, MMP-9, Cyclin D1 and Bcl-2 was increased when TAZ was upregulated. **C.** Regulation of TAZ on C-myc expression in glioma cells.

The expression of relevant biomarkers to cell proliferation, invasion, cell cycle progression and apoptosis, including Ki67, MMP-9, Cyclin D1and Bcl-2 was also detected and all these proteins in LN229 and SNB19 cells transfected with TAZ siRNA were downregulated, while upregulated in U118 and U87 cells transfected with TAZ-pcDNA3. Expression of C-myc was identified to be in accordance with the level of TAZ in LN229 cell and U87 cell (Figure 9A,B,C).

**Figure 10:**
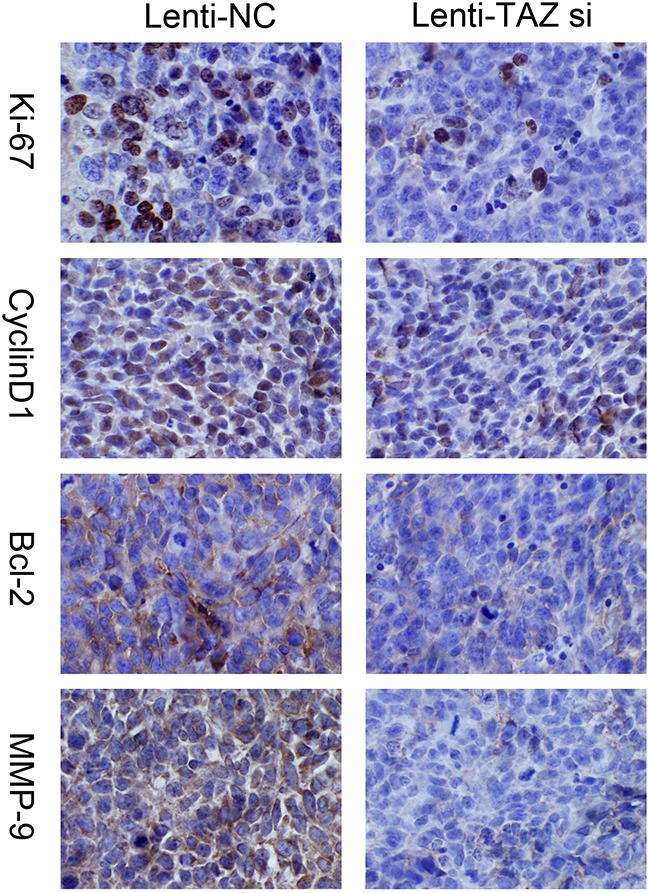
Expression of Ki67, MMP-9, Cyclin D1, Bcl-2 was suppressed in xenograft samples with TAZ knocked down examined *by* immunohistochemiscal staining (×200) Immunohistochemistry images showed that Ki67, MMP-9, Cyclin D1 and Bcl-2 expression were downregulated when TAZ was inhibited in xenograft samples.

We also detected the expression of Ki67, MMP-9, Cyclin D1, Bcl-2 in orthotopic xenografts by IHC, it was also found that the expression of Ki67, MMP-9, Cyclin D1 and Bcl-2 was downregulated when TAZ was knocked down (Figure 10).

**Figure 11:**
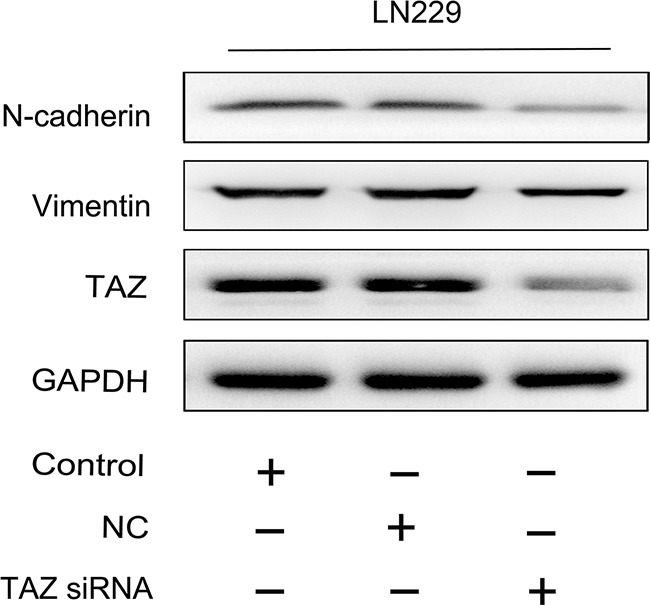
Vimentin and N-cadherin were suppressed when TAZ was knocked down in glioma cell Detected by western blotting, the expression of Vimentin and N-cadherin was decreased when TAZ was downregulated in glioma cell.

Since the promoting effect of TAZ on glioma cell migration and invasion was observed by transwell and scratch assay, we detected the expression of mesenchymal markers, Vimentin and N-cadherin, the result showed that they both were downregulated when TAZ was inhibited (Figure 11).

### TAZ is combined with TEAD4

**Figure 12:**
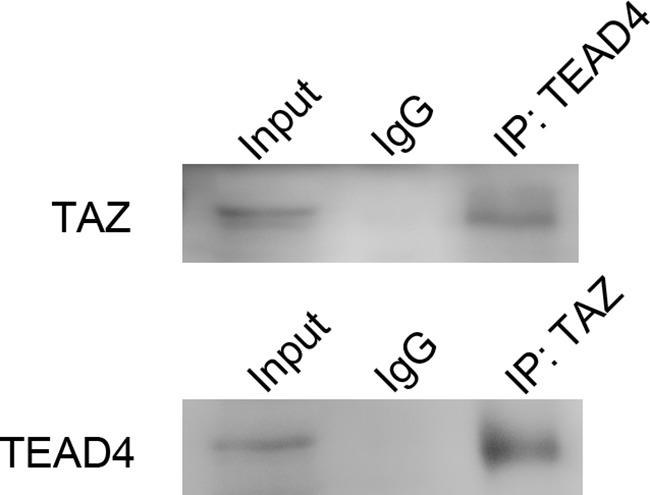
Co-immunoprecipitation of TAZ and TEAD4 Input lane was set as control to identify the target protein was comprised in precleared protein lysis. IgG lane was set as negative control. Anti-TEAD lane was protein precipitated by TEAD4 hybridizing with TAZ antibody. TAZ was detected. Anti-TAZ lane was protein precipitated by TAZ hybridized with TEAD4 antibody. TEAD4 was detected. The result of co-immunoprecipitation indicated that TAZ bound with TEAD4.

Immunoprecipitation of the binding proteins in the LN229 cells extracts with antibodies against TAZ and TEAD4 showed that TAZ interacted with TEAD4 (Figure 12). This result indicated that TAZ also bound to TEAD4 in glioma cells for regulating transcription and expression of target genes.

## DISCUSSION

Hippo signaling pathway is one of commonly mutated cancer pathways. TAZ and its paralog, YAP, the main downstream effectors, are suppressed by Hippo pathway, can be activated when Hippo signaling is dysregulated [3, 16]. TAZ is a transcriptional coactivator, without a DNA-binding motif, functions by transactivating a number of transcription factors, including TEAD, Runx2, SMADs, PAX8, TBX5 etc, and TEAD is a major facilitator of TAZ function. TAZ needs to pair with TEAD and induces the expression of target genes, i.e, several growth promoting factors and proapoptotic factors, such as c-myc, Cyr61, CCN1, TGFβ2, MCL-1 (myeloid cell leukemia-1), DDIT4, Trail and CTGF etc [17–20].

Aberrant TAZ expression has been reported in a variety of tumors. It has been reported TAZ is overexpressed in 20% of breast cancer specimens [7]. Positive expression of TAZ was observed in 121 of 181 (66.8%) non-small cell lung cancer (NSCLC) cases and was associated with poorer differentiation, metastasis stage, intratumoral vascular invasion, and poorer prognosis [11]. TAZ expression was high in pancreatic cancer tissue and positively correlated to tumor differentiation [21].

There are very few studies on deregulation of TAZ in gliomas. Li et al reported that expression of TAZ in glioma tissues was significantly higher than that in normal brain tissues [12]. TAZ has also been reported to have higher expression level in MES (mesenchymal subtype) glioblastoma (GBM) than in PN (proneural subtype) GBM, and complexes of TAZ and TEAD are recruited to most of MES gene promoters, when TAZ is silenced, MES markers, invasion and tumor formation are suppressed [22]. Besides, TAZ has been confirmed as the target gene of miR-130b and miR-125a-5p in glioma [23, 24].

Our results show that TAZ is markedly overexpressed in most of malignant gliomas, and its expression rate and level are positively correlated to tumor grade, which suggests that TAZ may be associated with the development and progression of gliomas. IHC, IF staining and Western blotting have been performed to detect the subcellular localization of TAZ in glioma cells, the results demonstrate that the expression of TAZ in both cytoplasm and nucleus of tumor cells is increased, whereas its upregulation in nucleus is more significant than that in cytoplasm and increases with the ascending order of tumor grade. This finding indicates that when the Hippo pathway is inactivated, its downstream effector TAZ is translocated to the nucleus and induces the expression of a variety of proteins implicated in cell growth and apoptosis.

We further knock down and overexpress TAZ in glioma cell lines *in vitro* and show that cell proliferation, invasion and migration are inhibited, cell cycle progression is arrested, and apoptosis is induced when TAZ knocked down. The opposite results are observed when TAZ is overexpressed.

In orthotopic glioblastoma mouse model, it has been identified that tumor growth is suppressed and survival is extended when tumor cells knocked down TAZ are implanted. The expression of biomarkers relevant to cell proliferation, invasion and apoptosis, such as Ki67, Cylcin D1, MMP9 and Bcl-2 has coincident change with knockdown or overexpression of TAZ.

As a vital transcription regulator, C-myc is associated with 20% of human cancer, and plays an essential role in the regulation of many physiological processes including cell cycle control, apoptosis, proliferation and cell adhesion. C-myc has been reported as the target gene of TAZ and TEAD [17, 25]. We have shown that the expression of C-myc is consistent with the level of TAZ, so TAZ might promote glioma progression through activation of C-myc.

TAZ has been reported to contribute to EMT in many tumors. TAZ is in a complex with TEAD2, directly recruited to a majority of EMT gene promoters, like ZEB1 [26]. EMT is associated with the acquisition of migration and invasiveness. In this study, TAZ has been identified to exert effect on promoting invasion and migration of glioma cells. Meanwhile, the mesenchymal marker: vimentin and N-cadherin [27] are downregulated when TAZ is knocked down.

TAZ has no DNA binding domain, but has a N-terminal TEAD-binding motif. It regulates transcription of target genes by interaction with DNA binding transcription factors. The major TAZ interacting transcription factors are TEADs. In mammals, there are four members, TEAD1-4, in TEAD family. TEADs act as important effectors of TAZ to stimulate cell proliferation, migration and epithelial-mesenchymal transition [28]. In glioma, only TEAD2 has been reported to bind with TAZ to regulate transcription of a majority of mesenchymal genes [22], while TEAD4 has not been reported to bind with TAZ in glioma cell up to now. As shown in Co-IP we have found that TAZ also bind to TEAD4 in glioma.

In conclusion, TAZ expression is upregulated in glioma cells and positively correlated to tumor grade, mainly localized in nucleus in high grade gliomas. TAZ promotes glioma cell proliferation, inhibits apoptosis, enhances cell invasion and migration, and contributes to EMT in glioma cells. The results imply that TAZ plays an important role in gliomagenesis and may present as a potential target for glioma therapy.

## MATERIALS AND METHODS

### Tissue specimens

Three nontumoros brain tissues and 41 glioma specimens were obtained during surgical resection from patients at Tianjin Medical University General Hospital during 2012~2014. The histopathological diagnosis and grading of glioma specimens were assessed according to The 2007 WHO Classification of Brain Tumorsby two neuropathologists independently. Glioma specimens consisted of 3 cases of pilocytic astrocytoma (Grade I), 8 cases of astrocytoma (Grade II), 5 cases of oligodendroglioma (Grade II), 6 cases of anaplastic astrocytoma (Grade III), 3 cases of anaplastic oligodendroglioma (Grade III) and 16 cases of glioblastoma (GBM, Grade IV). Three samples of nontumorous brain tissue were removed during intracranial decompression surgery. Part of each specimen was snap-frozen and stored at liquid nitrogen, and the remainings were fixed with 4% formalin for histopathological and immunohistochemical examination. All the procedures were approved by Institutional review board of Tianjin Medical University Medical Principle Committee.

### Cell culture

Seven human GBM cell lines (U87/LN229/SNB19/U118/A172/U251/LN308) were obtained from China Academia Sinica Cell Repository (Shanghai, China). Another TJ899 GBM cell line was obtained from Laboratory of Neuro-Oncology, Tianjin Neurological Institute. All cell lines were maintained in Dulbecco's modified Eagle's medium (DMEM, Gibco) supplemented with 10% fetal bovine serum (FBS), and maintained at 37°C in 5% CO_2_.

### Real-time PCR analysis

Total RNA was extracted with Trizol (Life technology, USA), and reverse transcription was performed using M-MLV reverse transcriptase (Promega, USA) according to the manufacturer's protocol. Real-time PCR was performed using SYBR Green Master mix as the manufacturer's instructions. Expression levels of GAPDH were used for normalization and quantification of TAZ expression levels. Real-time PCR data were analyzed by the 2^ΔΔCt^ method. The following primer sequences were used: TAZ (F:5′-AGT ACC CTG AGC CAG CAG AA-3′; R:5′-GAT TCT CTG AAG CCG CAG TT-3′), GAPDH (F:5′-GGT GAA GGT CGG AGT CAA CGG-3′; R:5′-GAG GTC AAT GAA GGG GTC ATT G-3)′.

### Western blot analysis

Total cell lysates were prepared using RIPA lysis buffer added PMSF (Solarbio, China). Cytoplasmic and nuclear TAZ protein were extracted separately using nuclear and cytoplasmic extraction reagent kit (Beyotime Bio Corp, China). Protein concentration was determined using BCA protein assay kit (Beyotime Bio Corp, China). Proteins (20 μg) were resolved by SDS-PAGE and electrotransfered to PVDF membranes (Millipore, USA). Appropriate primary antibodies (Santa Cruz, CA, USA) were used for WB analysis.

### IHC and IF staining

For IHC, incubated with appropriate primary antibodies (1:100 dilution), sections were then incubated with secondary antibody and diaminobenzidine (Zhongshan Bio Corp, China), counterstained with hematoxylin, and visualized under light microscope. For IF, sections were incubated with the primary antibody and FITC-labeled secondary antibody. DAPI reagent was used to stain the nuclei and visualized using FV-1000 laser scanning confocal microscopes and analyzed using IPP5.1 (Olympus, Japan).

#### Oligonucleotides and plasmid transfection

The TAZ and scramble siRNA were chemically synthesized with the following sequences: scramble sequence: 5′-UUCUCCGAACGUGUCACGUTT-3′ and TAZ siRNA: 5′-GGAUACAGGAGAAAACGCATT-3′(GenePharma, China). The recombinant plasmid TAZ-pcDNA3 was constructed by Genscript Co. Ltd, China.

The oligonucleotides and plasmid were transfected into cells at 70% confluence using Lipo3000 according to the manufacturer's instruction (Invitrogen).

#### Cell proliferation assay

SNB19, LN229, U87 and U118 cells were seeded into 96-well plates at 4000 cells per well. After transfection as mentioned above, MTT assay was used to determine the cell viability in consecutive 5 days as previously described [13].

The data are presented as the mean±s.d, which are derived from triplicate samples of at least three independent experiments.

#### Cell cycle analysis

For cell-cycle analysis using flow cytometry, transfected and control cells in the log phase of growth were harvested, fixed with 90% ethanol, then incubated with RNase. Nuclei of cells were stained with propidium iodide. A total of 10^4^ nuclei were examined in FACS Calibur flow-cytometer (Becton Dickinson, NJ, USA). DNA histograms were analyzed using Modifit software. Experiments were performed in triplicate. Results are presented as percentage of cells in each phase of cell cycle.

#### Scratch assay

2 × 10^6^ cells were seeded on 6-well plates and cultured overnight. When the cells were 80% confluent, a straight scratch was gently made through the central axis of the plate using a micropipette tip. The plates were rinsed with PBS solution then the serum-free DMEM was added and incubated for additional 24hr. Five randomly selected visual fields were visulized under an inverted microscope (Olympus, Japan) at stage of 0 and 24h incubation to measure the width of the scratched gap by Image J software. The results were shown as the closure percentage of gap at 24h in comparison with 0h, using following formulae as described before [14]:

$$\text{Gap closure}(\%)=\frac{{\text{Gap}}_{0h}-{\text{Gap}}_{24h}}{{\text{Gap}}_{0h}}$$

where Gap_0h_ = Gap width at 0h

Gap_24h_ = Gap width at 24h

The result of experiments was statistically analyzed.

#### Transwell assay

Transwell filters (Costar, USA) were coated with Matrigel on the upper surface of the polycarbonic membrane. Transfected and control cells suspended in 200 μL of serum-free DMEM were added to the upper chamber and conditional medium of NIH3T3 cells was placed into the lower chamber as a chemo-attractant. After 24 hr of incubation, the medium was removed from the upper chamber. The non-invaded cells on the upper surface of the inserted filter were gently scraped off with a wet cotton swab. The cells that had invaded the lower surface of the filter were fixed with 4% paraformaldehyde and stained with hematoxylin. The migrated cells were counted by light microscopy and the average number of cells of at least three fields from each well was calculated.

#### Apoptosis assays using annexin staining and TUNEL

Parental and transfected cells in the log phase of growth were harvested and collected. For the Annexin V assay, the annexin V-Cy3-labeled Apoptosis Detection Kit (Abcam, USA) was used. The apoptotic cells were detected and quantified using FACSCalibur (Becton Dickinson, USA). The data obtained were analyzed using CellQuest software. The apoptotic cell death in the tumor specimens of nude mouse models from *in vivo* study was examined by TUNEL method using in situ cell death kit (Roche, USA). Nuclei were counterstained with DAPI karyotyping kit (Genmed, China) and visualized using FV-1000 laser scanning confocal biological microscope and analyzed by IPP5.1 (Olympus, Japan).

### Co-Immunoprecipitation (Co-IP)

Protein of cultured cells was extracted, the lysate was precleared by adding control IgG (Beyotime Institute of Biotechnology, China), together with Protein A/G plus Agarose (Santa Cruz, USA). After centrifugation, the supernatant was regarded as precleared protein lysis and added with the relevant primary antibodies, then incubated with Protein A/G plus Agarose on rotary shaker overnight. Immunoprecipiatates were collected by centrifugation and gently washed with PBS. The bound proteins were resuspended with loading buffer and analyzed using Western blotting.

### Orthotopic glioblastoma mouse model implanted LN229 cells transfected with TAZ siRNA

This *in vivo* study was approved by the ethics committee of Tianjin Medical University, General Hospital.

The 6-week-old male balb/c nude mice were randomly divided into Lentivirus with TAZ siRNA (Lenti-TAZ si) and Lentivirus with scramble sequence (Lenti-NC) (Genepharma, China) groups with each group being consisted of six mice. The mice of Lenti-TAZ si group were implanted with 5 ×10^4^ LN229 cells transfected with Lenti-TAZ si to the brain with a stereotactic instrument using a guide-screw system as previously described [15], whereas the mice in the Lenti-NC group were implanted with 5 ×10^4^ LN229 cells transfected with Lenti-NC.

*In vivo* bioluminescence imaging (BLI), was examined regularly to observe the tumor growth. Meanwhile, the overall survival of mice in both groups was observed. For imaging, the mice were injected intraperitoneally with D-luciferin (Promega), and imaged with the IVIS Lumina Imaging System (Xenogen Corp, USA). Tumor tissues were removed for formalin fixation and preparation of paraffin-embedded sections at the end of observation.

### Statistical analysis

Data were expressed as means±s.e. Statistics was determined by ANOVA, χ^2^ test, or Student's t-test using SPSS11.0. Statistical significance was determined as P<0.05 (*).
